# Hysteria

**Published:** 1835

**Authors:** Charles Woodward

**Affiliations:** Cincinnati


### IV.—Hysteria.

*Observations and Speculations on the Pathology of Hysteria; read before the Cincinnati Medical Society.* By Charles Woodward, M. D.

Mr. Tate, in his late work on hysteria, has given us a new diagnostic sign of this malady; one which my own limited experience induces me to believe is of considerable value. I refer of course to his assertion that tenderness evincive of spinal irritation, is always present with hysterical symptoms. It is, however, I think, incorrect that uterine derangements are always present in this malady, as he affirms. In this sweeping declaration, he is certainly opposed by the experience of the profession;—for who has not had the most preplexing cases of hysteria where the menstrual secretion, if we can depend upon the statements of our patients, has in no respect

4 *

been defective,—where, indeed, no uterine irritation of any kind has been discovered upon the closest scrutiny. Again, who has not observed derangements, organic and functional, of the most serious character, affecting this very organ, without the nervous system being called upon to sympathize in any perceptable manner, or at least in any degree proportionable to that which is supposed to excite the moral and physical derangements, which at times characterize hysteria. The idea that nervous diseases are *essentially* connected with disorder of the uterine functions, is not novel. Some writers of considerable antiquity have expressed the same opinion; and, of consequence, many gratuitous powers were attributed to the organ by them. Many respectable authors of more modern times had maintained the same hypothesis, long before Mr. Tate favored the world with his opinions; so that to deny the *occasional* connection of this organ with disease of the nervous system, would be as preposterous, as, with Mr. Tate, to assert its universality. Indeed, with Dr. Good, we readily admit, that with a morbid condition of the uterus, hysteria is in many instances very closely connected, though it is going too far to say that it is always dependent on such a condition, for we meet occasionally with instances, in which no possible connection can be traced between the disease and this organ, and sometimes witness it in males as decidedly as in females. Denying then, as we do, to the uterus, any *specific* power in the *production* of those diseases, which by common consent are denominated *nervous*, we are forced to conclude that to the brain and spinal cord, more rationally than to any other part of the system, are ascribable those mysterious and sudden metamorphoses, which invariably, in a greater or less degree, attend or constitute the disease under consideration. Changes which ever have, and probably ever will, bid defiance to any attempt at nosological arrangement.

The probability of this opinion is supported by what little is actually known of the influence exercised by the nervous system, over the vascular and muscular functions, though to attempt to fix the degrees of diseased action in the brain and spinal marrow, by any inference drawn from the innumerable forms of disease simulated by hysteria, would only be time lost.

Fortunately for society, this affection, often so truly distressing, rarely terminates fatally; and thus, but very few opportunities are afforded, by post mortem examinations, for drawing correct inferences on the true nature of the disease. Modesty, therefore, requires the acknowledgement, that but little is certainly known concerning it. In fact, from the rare mortality of these affections, we can have no standard of comparison, by which to estimate the condition of the brain and spinal marrow, in various nervous complaints. Indeed, the complex relations of the nervous system taken in connection with our moral as well as physical nature, must ever remain the subject of curious yet uncertain speculation; and, conseqently, our views of hysteria must continue in a great degree unsatisfactory.

It will be observed, from these remarks, that I consider spinal or encephalic irritation, however obscurely presented to our observation, as essential to the phenomena of hysteria; and that the uterine functions are not unfrequently perverted, either in the relation of cause or effect. The first proposition is in accordance with the views of Mr. Tate, and requires no further consideration; the second, however, denies his position of the universality of uterine derangement, and confines it to an occasional occurrence.

If, as Mr. Tate ingeniously argues, the disease is uniformly dependent upon uterine derangement, the fact quoted above from good authority, that it occurs occasionally in males, remains unexplained. This fact might perhaps have yielded to the weight of evidence adduced by Mr. Tate, if we had the authority of Good, *alone*, to oppose to it; but when upon reference we find that Cullen, and Caldwell, and Dewees, with other illustrious names may be brought forward to sustain us, we feel safe in calling upon the gentleman for a stronger and more extended evidence, before we can acknowledge our conviction of the correctness of his pathology. Cases could easily be adduced from the experience of every practical physician, which would conclusively disprove this fundamental proposition in the useful little work to which we have alluded. But it may, perhaps, with more plausibility be urged by the supporters of the opinions of Mr. Tate, that in the spirit of opposition, we have confounded *hypocondriasis* with *hysteria*, and have carelessly or disingenously overlooked the *diagnostics* of these two somewhat similar affections, or, in other words, that we have improperly attributed to a hysterical condition of system, that unpleasant chain of morbid nervous association, which only characterizes hypocondriasis. It is true that both these diseases may very properly be classed among nervous affections, and that to a superficial observer, their slight resemblance may be considered sufficient to establish their identity, but in truth their distinctive features present too broad a line of demarkation to be so mistaken, if due attention be paid to them. The diagnostics are, in fact, simple and few, and to identify them would be the result of ignorance, or of that blinded enthusiasm, by which favorite theories, however absurd, are occasionally supported. It may be well to present a few of their diagnostic peculiarities. 1st. Though spasmodic affections are liable to occur in both of these diseases, it will be admitted they are neither so frequent or so severe in hypocondriasis as in hysteria. 2nd. Persons liable to hysteria are sometimes affected with indigestion, though more frequently they are entirely free from any gastric or hepatic affection. Hypocondriasis is strikingly characterized by functional derangement of the stomach and liver, and not unfrequently by structural lesions of both. 3rd. But the constitutional temperament, and the period of life of the occurrence of these complaints afford, also, very important grounds of difference. Hypocondriasis generally affects those of bilious and melancholic temperament—hysteria, the sanguine and plethoric:—the first rarely occurs very early, or the last very late in life—the first becomes aggravated, the latter relieved by an advance in years.

These few distinguishing peculiarities ought to prevent the possibility of confounding the two forms of disease, or at least shield us from the imputation of ignorantly identifying them, in the opposition we have manifested to the opinions of Mr. Tate, in reference to the universality of uterine disease. We are, indeed, forced to the conclusion that he has viewed the subject in a manner entirely *ex parte*, and that for the sake of supporting his theory, he has neglected to report cases calculated in any degree to militate against his favorite hypothesis. This part of the subject has been thus exhibited, from the conviction that, by locating the disease under consideration uniformly in the uterus, he has attributed to that organ a sympathetic power over other parts of the system which it in no respect possesses, and that he has deprived the brain and spinal cord of that physiological importance which they certainly deserve, in consequence of the acknowledged influence they exercise over every moral and physical faculty. It is true, no direct evidence can be presented, that the brain and spinal marrow are the seats of hysteria, for the disease rarely proving fatal, opportunities of dissection, as already remarked, are by no means frequent. Even Dr. Dewees declares that he has no recollection of ever having observed a *post mortem* report of this disease, in which the condition of the brain was particularly noticed; and on the same subject he proceeds to quote the observation of Dr. Whytt, that although it appears from dissection of those who have died of nervous affections, that the stomach and intestines, liver, spleen, *omentum*, messentary or *uterus*, have frequently been found obstructed, scirrhous, or otherwise unsound: yet, in as many other cases, of the same disorders, no such morbid appearances have been observed after death.

It follows, that these symptoms may frequently proceed from causes which, eluding our senses, are not to be discovered by dissection. Nay, obstructions, scirrhi, and other disorders of the viscera, are observed in those who have died after suffering long from nervous ailments, seem sometimes to have been the consequence of a long state of bad health, rather than the causes of it. With these difficulties in the way of a correct pathological inference, we feel the better satisfied to rely for the establishment of our views of this disease upon what evidently appears to be its *remote and exciting causes*, and upon the singular phenomena which it almost uniformly presents.

We have already referred to the brain and its appendages, as the true seat of hysteria, and though we cannot demonstrate the peculiarity of the change, which, from the variety and violence of the symptoms, we are forced to believe exists; still we think we are justified in our conclusions, from a careful examination of the phenomena which are manifested in the progress of the disease.

The observations of Dr. Samuel Jackson, on this subject, are clear and expressive of our own sentiments:

"The brain," he remarks, "is a collection of organs, of nearly similar composition, which preside over the various intellectual and pathetic faculties, and voluntary motion. The medulla oblongata appears to be the central organ of perception, and volition, and its lower portion, and the upper part of the spinal marrow, govern the expressions and the respiratory muscles. Irritation of the upper portion of the medulla, occasions spasms, convulsions, etc., of the voluntary muscles, and of the lower portion, irregular and spasmodic contractions of the muscles of respiration, of the voice, and of the face, as expressing the passions. Hence the sense of suffocation, sighing, screaming, laughing, crying, weeping, coughing, and the various distortions of the countenance. Individuals who experience frequent attacks of hysteria, have this portion of the central structure in a permanent state of irritation, of feeble grade, and which is increased by any sudden and strong impression, an unexpected noise, sight, or intelligence, becomes in them an exciting cause of hysteric paroxysms. Venereal irritation, sufficiently intense to be transmitted to the brain, is often communicated to its central organ, and excites the symptoms of hysteria. The stomach and uterus are those parts from which this irritation is most commonly transmitted, and is effected through the *great sympathetic*, which anastomoses with the pneumo-gastric, or par vagum, that has its origin in the medulla oblongata."

The nerves, to a morbid condition of which Dr. Jackson very properly refers most of the phenomena of hysteria, are such as connect the internal organs of respiration with remote parts. The respiratory nerves have no ganglia at their origins; they come off from the medulla oblongata, and the upper part of the spinal marrow; and from this origin they diverge to the numerous muscles and other parts, which are combined in the motions of respiration, although they are supplied with nerves from other sources.

Among these nerves, as more immediately connected with the subject before us, may be enumerated—1st. The par vagum, or pneumo-gastric nerve; 2d. The respiratory nerve of the face; 3d. Superior respiratory nerve of the trunk; 4th. Great internal respiratory nerve, (phrenic or diaphragmatic;) 5th. The external respiratory nerve; and, 6thly. As connected in a greater or less degree with all the rest, the great sympathetic or ganglionic.

Now when the portions of the medulla oblongata and spinal marrow, whence these nerves arise, are morbidly affected, the parts upon which they are distributed become disordered in their functions, whether the remote cause was moral or physical in its nature, or acted primarily on the nervous centre, or on some distant part. It is well known that when the passions or emotions of the mind are too actively excited, the liver and stomach are liable to be seriously affected, the one to the more abundant secretion of bile; and the other to the effort of throwing it off. On the contrary, when from any cause, the mind is depressed by melancholy, the liver ceases in a great measure its important functions, the bile is not duly elaborated, the bowels consequently become torpid, and the stomach dyspeptic, and these disordered organs, reacting through the medium of their nerves, upon the cause of their own derangements, increase the evil, and involve the unhappy subject in deeper ruin.

Hence the danger of mere moral affections becoming in time decidedly physical, unless suitable means are instituted to destroy the chain of morbid association. This condition of things not unfrequently attends hysteria, until a slight nervous irritation terminates in the most inveterate functional disorder, and even proceeds to structural derangement of an altogether incurable character. When this is the case, revealed as it is, by post mortem examination, the effect often passes for the cause, and incorrect pathological views are consequently entertained. The powerful sympathy existing between the brain and the uterus, after the female has arrived at adult life, becomes in some constitutions a constant source of irritation. The two organs are capable of acting and reacting upon each other, in a manner truly anomalous, for we find between no others a sympathy proportionate to this; we need not therefore be surprized, that uterine irritation, whether excited by excessive venereal orgasm, by exposure to atmospheric vicissitudes, or by any other physical or moral cause, in *persons predisposed to hysteria*, should, by extending its influence through the great sympathetic, to the brain and respiratory nerves, increase the general irritability of the system, and give rise to all the aggravated phenomena discovered in the vascular, muscular, glandular, and lymphatic systems. We believe this is *not unfrequently* the case, where we find the menstrual secretion defective in any respect, in nervous temperaments; but that this is to be esteemed the correct pathology of hysteria, *uniformly*, is not only at variance with the experience of every practical member of the profession, but is also at war with the long catalogue of symptoms which often exist in hysteria, *independently* of any perceptible deviation from health in the uterus. As before remarked, the brain and its appendages have a most extensive chain of sympathies, every one of which can in a greater or less degree contribute to the formation of the symptoms characterizing this complaint. If, for instance, from any cause, the *gastric filiments* of the par vagum, are morbidly affected, so as to become the principal seat of sympathy, we shall have a chain of gastric symptoms presenting themselves, such as eructations, sour belchings, gastrodynia, pyrosis, indigestion, and the globus hystericus, without there being necessarily a single sympathetic or associated action with the uterus. If the nervous branches from the great sympathetic, which supply the intestines, become the concentrated point of nervous irritation, we may find tympanites, spasms, diarrhœa, or costiveness, with painful and sometimes spasmodic contractions of the abdominal muscles. If the hepatic plexus becomes, as is very often the case, in melancholic constitutions, the point of diseased sympathetic action, we shall find the liver essentially disordered, the secretion of bile, being either diminished, increased, or in some manner vitiated and unfit for the purposes designed: pain in the right side, with an unpleasant sense of fulness, and, where the irritation has been of very long continuance, structural derangement ensues, biliary calculi are found in the gall bladder, and sometimes extensive lesions of the organ itself, terminate the sufferings of the patient. This condition of the liver, and the nerves supplying it, is, we believe, more frequently dependent on that state of the brain which constitutes melancholic, than hysteria; several of the enumerated symptoms, may with equal propriety mark some of the more aggravated shades of hysteria.

If the nervous fibres* supplying the kidneys become the medium of an irritative sympathy, we have an immoderate flow of pale or limpid urine; a symptom, by the by, which is *often* presented to our attention in these perplexing complaints: sometimes, however, the secretion is diminished, of a high color, and very offensive. Bloody urine is occasionally evacuated, and a pain in the part is sometimes felt, resembling the passage of calculi along the ureter. These extreme symptoms, and especially the bloody urine, we believe are presented only when the sympathies have existed long enough to have established organic derangements which may exist independent of the original cause.

Other parts of the body, as the heart, the bladder, and the scalp, are points of morbid sympathy, and present to the observing physician the symptoms which might be anticipated from diseased action interrupting the healthy performances of the functions of these important parts of the human economy. When the nerves of the heart are more particularly called upon to participate in the nervous derangement, we perceive palpitations which at times are exceedingly harrassing to the unhappy sufferer. If the bladder become the point of morbid sympathy, we occasionally find retention or incontinence of urine, and mucous discharges. We have not been called upon to witness these forms of the disease, but we should presume, if not quickly counteracted, they might assume a dangerous aspect, and defy the ordinary routine of practice.

Of the last form to be mentioned, we have seen but one instance, and that was truly the most perplexing case of hysteria we have ever been called upon to contend with. We allude to the affection of the scalp. This form is said to be attended with a coldness on the top of the head, or a sense of heat on the back part, great tenderness to the touch, etc., etc. In the case now alluded to, besides the above symptoms, we observed a spongy puffiness of the top and back part of the head, with a manifest *pointing* at the vertex. Such were the symptoms presented by a young lady about 17 years old, by whom I was consulted in the fall of 1827. Hitherto no hysterical paroxysms had been observed, no catarmenial derangements were discoverable, in a word, but for the singular appearance presented by the scalp, she would have been pronounced in good health and far from the probability of disease. She had a most luxuriant crop of hair, to the ornamental character of which she was by no means unconscious, and with female nicety she braided and tied it, so as to draw upon the stretch the affected part of the integuments. To this I at first attributed the disease, and as there was apparently a strong tendency to suppuration, I removed a part of the hair, and applied poultices. In a few days from the *appearance* of fluctuation, I punctured the swelling with a lancet. No purulent matter followed, and my patient immediately fell into a profound coma, from which in a few hours she was roused, only to become the victim of the most distressing series of convulsions I have ever witnessed. These painful scenes were renewed almost daily for weeks and months, withont any decided alleviation from the long list of pharmaecutic agents which were suggested by my own mind, and some of the most practical men of the profession. Neither my consulting physicians or myself, suspected the true character of the disease, but from its obstinacy our worst fears were excited, lest some cerebral irritation of an incurable character existed.

By one gentleman of considerable surgical eminence, it was proposed to lay open the scalp by a free incision, and even to trephine the skull, to discover and drag to light the hidden enemy. To this I did not consent, but finding but little assistance in the advice of my consulting friends, I ordered the whole scalp to be shaved, and the Tart. Emetic ointment to be freely used over the head and back of the neck. When the pustular effect was freely and painfully excited, the disease began to yield;—the syncope and convulsions with her mental disquietudes gave way, she regained a tolerably good share of health, married, and became a mother. Her constitution, however, had received a blow, from which I fear it will never entirely react.

The practice in this case was nearly the same with that of Mr. Tate, though it occurred long before his views were ever given to the public. But what he considers *essential* to the existence of hysteria was absent, and would probably have tended to embarrass his pathological conclusions, as it certainly did our own. Perhaps, however, his professional sagacity would have detected some slight shade of uterine derangement sufficient to have established his views which, to other and less skilled observers, would have remained entirely unobserved.

The nerves of certain organs are more liable than others to sympathize, with the irritations to which the brain and spinal marrow are, from a thousand inappreciable causes, exposed, and the phenomena of hysteria are always exhibited by the manifest derangement of those parts of the system which, in different constitutions, are most easily acted upon. Hence we find the stomach in one the seat of diseased action; the intestines, in a second; the liver, in a third; and the uterus, in a fourth, etc.

That the uterus is most commonly called upon, we think is sufficiently evident, from the frequency of the menstrual deficiencies by which hysteria is attended. But if uterine derangement is *uniformly* to be considered the *cause*, as Mr. Tate alledges, how can we explain the *spinal irritation*, which we believe always in a greater or less degree exists. Can we presume that this irritation of the uterus is necessarily communicated to the spinal nerves, let its grade of diseased action be what it may? If so, we might expect to find the same pain on pressure of the spinous processes in uterine derangement, without hysteria, as we find with it, a point which we conceive would scarcely find an advocate in Mr. Tate himself.

But, admitting the point here assumed, why does he apply his powerful irritants to the spinal column, before he removes the *cause* of the evil. He acknowledges that such irritations do succeed, before the depraved secretions of the uterus are restored to their healthy character, and he confessedly resorts to measures to effect *this* last result, *secondarily*, only to prevent a *return* of the symptoms; but this he does after the morbid association has been interrupted by the Tart. Emetic ointment, a fact quite sufficient to stagger our faith in his hypothesis. We have, however, admitted the importance of the diagnostic mark he has so judiciously enforced upon our consideration, and believe it to be entirely owing to an extension of the *irritating cause*, whatever that may be, which, (whether constitutional or perhaps local and accidental, but excited by a thousand varied means,) is propagated through the medulla oblongata, to the *great sympathetic*. This important agent in connecting and sustaining the sympathies of the more important parts of our physical constitution, being thus morbidly excited by its influence, as we have already seen, disorders the functions of the various organs, laboring itself under increased excitement, as well from a specific influence, as from the reaction of the organs affected by it, it is found while passing down the spinal column, to extend its sinister influence even to the neighboring integuments, imparting that painful sensation when slight pressure brings these integuments into contact with the resistance of the spinous processes. "What renders it more probable that the brain, or at least the origin of the nerves of this organ, is the seat of that condition which gives rise to the convulsive motions in hysteria, are the experiments and observations of Dr. Philip, in his "Enquiry on the relation between the nervous and sanguiferous systems." He says, "that neither mechanical nor chemical stimuli applied to the nervous system excite the muscles of voluntary motion, unless they are applied near the origin of the nerves and spinal marrow." Dewees, p. 472.

This fact, discovered by this ingenious experimentalist, goes not a little way to establish the above hypothesis, and enables us with somewhat more of pathological accuracy, to apply our external irritants, with the view of breaking in upon the chain of morbid associations, which we think so certainly exists. The practical illustrations of Mr. Tate will give us not a little confidence in testing candidly the principles he has so enthusiastically espoused. We ardently hope that a more systematic enquiry into the character and multiform features of this disease, than has as yet been instituted by the profession, will ere long lead to results the most happy to the community.

*Cincinnati, March*, 1835.

